# Values, Motives, and Organic Food Consumption in China: A Moderating Role of Perceived Uncertainty

**DOI:** 10.3389/fpsyg.2022.736168

**Published:** 2022-02-28

**Authors:** Sheng Wei, Furong Liu, Shengxiang She, Rong Wu

**Affiliations:** School of Management, Harbin University of Commerce, Harbin, China

**Keywords:** organic food, altruistic value, egoistic value, health concern, environmental concern, uncertainty, purchase intention

## Abstract

The present research attempts to understand the importance of altruistic and egoistic values in determining consumers’ motives and intention to purchase organic foods. Using the face-to-face survey approach, a total of 1,067 responses were collected from consumers in China. Data analysis was performed using a two-step structural equation modeling (SEM) approach, i.e., measurement and structural models. The findings indicated that both values influence the intention to purchase organic foods through the mediation of motives. Specifically, the altruistic value influences the environmental concern (EC), and the egoistic value influences the health concern (HC). Moreover, the perceived uncertainty negatively moderates a relationship between consumer HC and organic purchase intention while positively moderating a relationship between consumer EC and organic purchase intention. Several implications and suggestions are also discussed.

## Introduction

Emerging food safety incidents and environmental problems have increased global consumers’ attention to food quality, safety, and environmental friendliness (Central News Agency, 2013; Hsu and Chen, 2014; Teng and Lu, 2016; Molinillo et al., 2020; Rana and Paul, 2020). This phenomenon makes consumers more aware of possible pesticide residues in food, excessive use of environmentally harmful pesticides and chemicals, and the credibility of production methods (Fernqvist and Ekelund, 2014; Alzubaidi et al., 2021), which in turn arises as consumers doubts about modern agricultural cultivation methods and promotes the interest and demand for organic foods (Mondelaers et al., 2009). A gradual, yet, extensive growth has been witnessed worldwide in demand for organic foods (Sultan et al., 2020) with global sales posited to have crossed USD 90 billion in the past 20 years (Willer et al., 2020). Organic food is considered to have both nutritional and environmental benefits (Kushwah et al., 2019), which could be easily integrated with individuals’ personal values.

In the past decade, people have noticed the shift in consumers’ food preferences. Consumers are beginning to prefer organic food to industrially grown food as it is considered eco-friendly and healthier (Tandon et al., 2020). In the past, organic foods have been more popular with consumers in developed countries, but in the past decade, organic foods have witnessed a great revolution in developing countries such as China. Organic food consumption in emerging markets is growing faster than in Western markets (Nafees et al., 2022). Although the organic food industry in China is still at its nascent stage, the demand for organic food has increased dramatically in recent years. With a continuous improvement of living standards, China is evolving into the fastest growing organic food market in the world. According to the ‘‘2020--2025 China Organic Food Industry Market Panoramic Survey and Investment Value Assessment Advisory Report’’ issued by China Research Institute, China will become the fourth largest consumer of organic foods, which are expected to account for 1--1.5% of the whole Chinese food market.^1^

Organic food consumption belongs to sustainable consumption and turning to organic foods is believed to help promote the sustainable development of consumption (Reisch et al., 2013; Feil et al., 2020). At the same time, organic food is also classified as ethical consumption because it shows concerns about the ecological environment (organic foods have less harmful effects on the environment) and is also beneficial to individuals (health). Several researchers have highlighted the importance of values in studying pro-environmental behavior or ethical behavior (De Groot and Steg, 2009; Hidalgo-Baz et al., 2017; Ahangarkolaee and Gorton, 2020; Vega-Zamora et al., 2020). Value is defined as “a desirable trans-situational goal varying in importance, which serves as a guiding principle in the life of a person on other social entity” (Schwartz, 1992). The literature shows that the altruistic value (concerning others) and the egoistic value (pro-self) are the two key driving forces for people to make moral behavior decisions. However, a direct prediction effect of values on individual behavior is weak (Homer and Kahle, 1988) and there is no consensus on the effect of consumer values on organic food consumption in the existing literature (Van Doorn and Verhoef, 2015; Yadav, 2016). In China, the studies related to organic food consumption are far from enough (Thøgersen et al., 2015; Teng and Lu, 2016; Lin et al., 2021; Liu et al., 2021). Most studies have focused on the consumer motivation to buy organic products and their values. Due to the vague boundary of the two concepts, most studies are separated (Kareklas et al., 2014; Teng and Lu, 2016) or mixed together (Yadav, 2016) when examining the effect of values or motivation on organic purchase. Jolibert and Baumgartner (1997) pointed out that the inherent values of society will form a definite behavioral motivation, and there was a hierarchical relationship between them. However, a few scholars in the field of organic consumption explore how consumer values drive consumption motivation, thereby affecting the purchase intention of organic foods. Although a few studies have adopted the causal chain perspective of “Values-Motivation-Behavior” (VMB) (Zhang, 2008; Gao et al., 2014), it is still a lack of application in the field of organic food consumption. Therefore, this study aims to investigate the mechanism of “VMB” in the context of Chinese organic consumption.

Further, there may be some obstacles in the influence of values and motivation on consumer behavior. Even if the main effect of values and motivation on organic food purchase behavior is clear, it is not enough to understand consumers’ final behavior. Therefore, another task of this study is to explore the moderation effect of perceived uncertainty on this influence process. In reality, consumers may lack the knowledge of organic foods, and the credibility of organic food-related certification is low, which will lead to consumers’ feeling of uncertainty. At present, due to various certification systems and food labels, the information credibility and standards of organic products are in a state of confusion, especially in China, which increased the difficulty to distinguish the information. In 2018, a survey of 2,006 respondents was conducted by the social survey center of China Youth Daily showed that 86.0% of the respondents had bought organic foods, and 50.5% of the respondents could not distinguish the foods which were organic. Around 69.9% of the respondents felt that ‘‘organic food’’ is more in name than in reality.^2^ In view of the current situation of an organic food market in China, it is necessary to study and understand the moderating role of perceived uncertainty when investigating the impact of consumer motivation on organic food purchase intention.

In sum, the multi-purpose of this study are as follows: (1) to explore the influence of values and consumption motives on organic purchase intention in the context of Chinese consumers; (2) to examine the mediating effect of consumption motives on the relationship between values and organic purchase intention; and (3) to examine the moderating effect of perceived uncertainty on the relationship between consumption motives and the purchase intention of organic foods.

## Literature Review and Hypothesis

### Consumer Values

Schwartz Value Theory is often used to study the relationship between values and consumer behavior (Schwartz, 1992). A few studies have proven the influence of values on pro-environment behavior such as the reduction of household carbon dioxide emissions (Steg et al., 2005), waste recycling, and resource utilization (Milfont et al., 2010), and green consumption behavior. For example, Homer and Kahle (1988) point out that the value dimensions will affect the natural food purchase. Kim and Kim (2010) propose that Korean consumer values affect the choice of green products through their attitude toward the environment.

The theory of Value-Belief-Norm (VBN) puts forward the egoistic value, altruistic value, and biosphere value, which are the main values related to environmental problems and can predict specific environmental behavior (Stern et al., 1999; Stern, 2000; Prakash et al., 2019). Altruistic values describe the situation under which peoples act on behalf of others while expecting no personal benefits (Schwartz, 1968). Instead, egoistic values mean acting on behalf of oneself or expecting personal benefits such as eliminating the suffering and harm of oneself (Kollmuss and Agyeman, 2002). For organic consumption, egoistic values mean that consumers want food to be beneficial to their health, while altruistic values mean consumers concerning no pollution to the environment. The research has considered both values as individuals’ consumption of organic food shows their concern toward the self-benefits as well as toward the environmental benefits (Kareklas et al., 2014). Yadav (2016) takes health concern (HC) and environmental concern (EC) as proxy variables of egoistic and altruistic values to investigate their impact on organic food purchase intention. ECs and HCs have always been the two most common motivations for organic food purchases (Wandel and Bugge, 1997). This study believes that values, as an abstract cognition existing in one’s heart, will have a positive impact on consumers’ motivation as well as purchase behavior intentions.

Values are the abstract expression of the subconscious, while motivation is a relatively concrete concept, which reflects an individual’s corresponding behavior or response stimulated by some factors. Motivation builds a bridge between values and behavioral intention, transforming abstract conceptual values into specific behaviors (Shen, 1994; Zhang, 2008). Accordingly, the hypotheses are proposed as follows:

H1a. Egoistic value positively influences an individual’s HC.

H1b. Altruistic value positively influences an individual’s EC.

H2a. Egoistic values among the individuals positively influence their intention to purchase organic foods.

H2b. Altruistic values among individuals positively influence their intention to purchase organic foods.

### Health Concerns and Environmental Concerns

Food safety, taste and freshness, environmental protection, and animal welfare are the common motivations of organic food consumption (Hemmerling et al., 2015; Rana and Paul, 2020). Among them, the health aspect is proved to be one of the two most prominent motivations for consumers to buy organic foods in Europe, Australia, Asia, and the United States (Schleenbecker and Hamm, 2013; Rizzo et al., 2020; Nafees et al., 2022). The importance of environmental preservation and food security is also found to be the main reason for buying organic foods in the Asian developing organic market (Sirieix et al., 2011; Teng and Lu, 2016; Ahangarkolaee and Gorton, 2020; Lin et al., 2021). Based on the review findings, the present study considers HC and EC as the dominant motives related to the decision of organic food consumption in a Chinese context.

The HC among individuals shows concern for self or to their family, so in nature, it is egoistic (Magnusson et al., 2003). HCs reflect the influence of egoistic values, that is, they want to keep themselves and their families healthy (Massey et al., 2018). Egoistic values drive consumers to pay more attention to health-related issues and then affect individual consumption decisions. Organic foods are considered to be healthier and more nutritious (Grankvist and Biel, 2001; Lea and Worsley, 2005) because they are produced without any harmful chemical fertilizers (Pino et al., 2012). When purchasing organic foods, health-related problems and safety concerns are considered as the main motivating factors (Goetzke and Spiller, 2014; Le-Anh and Nguyen-To, 2020). More specifically, the desire for body health and wellbeing drives the demand in food markets. Extensive empirical studies show that HCs are among the important drivers for developing a positive intention toward organic food consumption (Schifferstein and Ophuis, 1998; Kareklas et al., 2014; Yadav, 2016).

On the other hand, EC can be regarded as altruistic in nature because individuals are aware of the consequences of environmental problems and are willing to make efforts to protect the environment with little thought of benefits for themselves (Dunlap and Jones, 2002; Thøgersen, 2011; Wang et al., 2020). Personal concerns for the environment are related to consumers’ altruistic value or altruistic purchase considerations as consumers often choose organic food products due to consumers’ thinking that the production mode of organic foods does not harm the environment and has the attribute of ecological friendliness (Lee and Hwang, 2016). It has been suggested that the choice of organic foods over traditional food indicates consumers’ concern for others and the common interests (Thøgersen, 2011; Kareklas et al., 2014). EC shows the degree to which consumers are aware of environmental problems and support efforts to solve them or express their willingness to contribute personally to solve the problems (Dunlap and Jones, 2002). A growing body of literature on organic food consumption suggests that environmental concern plays a crucial role in influencing the consumption intention of organic foods as buying organic foods, which is considered to be a behavior beneficial to the environment (Kareklas et al., 2014; Hansmann et al., 2020; Liu et al., 2021). Accordingly, the hypotheses are proposed as follows:

H3a. HC positively influences the individual’s intention to purchase organic foods.

H3b. EC positively influences the individual’s intention to purchase organic foods.

H4a. HC mediates a relationship between egoistic value and organic purchase intention.

H4b. EC mediates a relationship between altruistic value and organic purchase intention.

### The Perceived Uncertainty

In the consumption scenario, uncertainty refers to the fact that due to information asymmetry, consumers face difficulty obtaining the complete product information or have doubts about the information obtained (Vieira, 2008), which will reduce the consumer’s purchase intention (Shiu et al., 2011). Some studies show that consumers tend to be skeptical about organic foods (Vermeir and Verbeke, 2006; Janssen and Hamm, 2011) as lacking the relevant knowledge or an insufficient understanding of organic labeling will produce perceived uncertainty (Janssen and Hamm, 2011). Additionally, consumers may be confused by the existence of multiple organic certification bodies because it is difficult to accurately evaluate the authenticity of organic certification, which also decreases consumer trust and further hinders organic consumption (Wang and He, 2016). Sometimes, eco-labels may send irrelevant, confusing, or even detrimental messages to consumers (Delmas and Lessem, 2017).

Prior research has indicated that perceived uncertainty negatively affects trust and the intention of purchasing organic foods (e.g., Yiridoe et al., 2005; Shiu et al., 2011). Especially, Vermeir and Verbeke (2006) emphasize the importance of mental processing uncertainty in organic food consumption. When consumers perceive a higher uncertainty, the more uncertain they are about the knowledge of organic foods, and the more difficult is to judge whether the organic food consumption decision is correct (Yiridoe et al., 2005). As Thøgersen (2009) suggests uncertainty may prevent people from purchasing organic foods even though they have favorable attitudes. Accordingly, even though consumers have strong motivations to buy organic foods, a higher level of perceived uncertainty may reduce the willingness to buy organic foods. In contrast, if the information about organic foods is credible, sufficient, and easy to obtain, the level of uncertainty may be reduced and a higher motivation will further strengthen consumers’ organic consumption behavior (De Magistris and Gracia, 2008; Teng and Lu, 2016). Therefore, it is speculated that perceived uncertainty will moderate a relationship between motives and organic purchase intention. Therefore, we proposed the hypothesis H5:

H5a. Perceived uncertainty positively moderates the relationship between HC and organic purchase intention. That is, when perceived uncertainty is higher, the relationship between HC and organic purchase intention is weaker and *vice versa*.

H5b. Perceived uncertainty positively moderates a relationship between environmental concern and organic purchase intention. That is, when perceived uncertainty is higher, the relationship between environmental concern and organic purchase intention is weaker and *vice versa*.

### Conceptual Model

**FIGURE 1:**
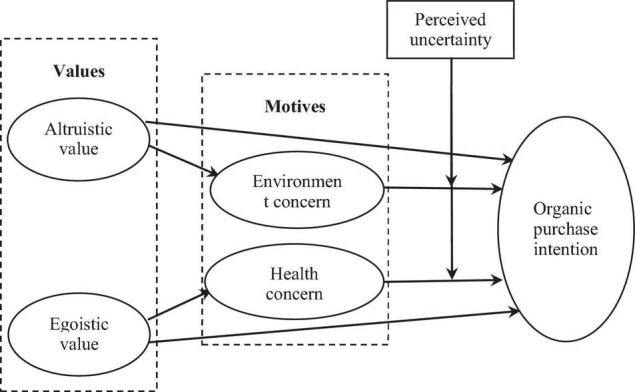
The hypothesized model for the present research.

It is important content in the field of consumer behavior to study the antecedents of consumer behavior intention from the perspective of values and motivation. However, in view of the vague boundary between the two concepts of values and motivation, existing studies usually only consider some factors and pay less attention to the complete causality from values to motivation to behavior. Zhang (2008) constructed the VMB model and then verified that values affect college students’ motivation to buy overseas study service products, and this motivation affects further purchase behavior. Drawing on the VMB model, Gao et al. (2014) explored the relationship among Confucian values, motivation, and TV purchase behavior of Chinese farmers. Recent studies have verified the driving effect of values on motivations, attitudes, and subsequent behaviors from different contexts (Hidalgo-Baz et al., 2017; Tarabashkina et al., 2020; Liu et al., 2021; Teah et al., 2021). Therefore, this study follows the same theoretical model, combined with the above literature discussion and hypothesis deduction, and constructs the following conceptual model (Figure 1) in the context of organic food consumption.

## Materials and Methods

### Measures

We designed a structured questionnaire to take a survey. The questionnaire includes two sections. In the first section, we measured the constructs. In the second section, we collected demographic information including gender, age, marriage, occupation, education, average monthly income, and the experience of organic food purchase.

In this study, multi-item scales are used to measure the constructs. To ensure the validity of the scale used in the survey, the items were adapted from the relevant research and existing literature. These scales are adopted from previous studies and modified to fit the theme and context of this study. The measurement items are backtranslated by native speakers to confirm that the contents and meanings are consistent with the original wordings. All items are assessed with the five-point Likert scales, ranging from 1 (“strongly disagree”) to 5 (“strongly agree”).

#### Values

The scales of Steg et al. (2005) are used to measure respondents’ altruistic and egoistic values.

#### Health Concern

The items of HC are from Yadav (2016). Example items include: “I chose food carefully to ensure the good health.”

#### Environment Concern

The construct of EC is measured by the four items borrowed from Yadav (2016). Example items include: “The balance of nature is very delicate and can be easily upset.”

#### Perceived Uncertainty About Organic Foods

A six-item scale deriving from the scale of Shiu et al. (2011) is used to measure perceived uncertainty about organic food knowledge, choice, and evaluation. Example items include: “organic food labels lead me to be unsure of the best choice for me,” and “I’m not confident of my personal view on organic food.”

#### Purchase Intention of Organic Food

Adopted from Steg et al. (2005), a five-item scale is used to measure the intention to buy organic food. Example items include: “I expect to consume organic food,” and “I would buy organic food products.”

### Data Collection and the Sample

In this study, a professional market research consulting company in China was entrusted to collect the data from January to March 2020. As the questionnaires were obtained by intercepting interviews at the entrance of the supermarkets located in Nangang District, Daoli District, Daowai District, Xiangfang District, and Songbei District of Harbin, convenience sampling methods were adopted. A total of 2,000 questionnaires were collected. After excluding 933 questionnaires with missing data, invalid answers, and subjects without organic food purchase experience, 1,067 valid questionnaires were retained. The demographic profiles of the sample are shown in Table 1.

**TABLE 1:** Demographic profiles of the sample (*n* = 1,067).

	*N*	%		*n*	%
**1. Gender**			**5. Occupation**		
Female	601	56.3	Student	205	19.2
Male	466	43.7	Civil servant	48	4.5
**2. Education**			Teachers, doctors and	155	14.5
High school or below	202	18.9	researchers		
College or university	696	65.2	Business operators	95	8.9
Postgraduate	146	13.7	Company employee	278	26.1
Missing	23	2.2	Worker	116	10.9
**3. Marriage**			Unemployed	14	1.3
Married	530	49.7	Housewives	17	1.6
Unmarried	487	45.6	Other	132	12.4
Divorce	36	3.4	Missing	7	0.7
Widowed	14	1.3	**6. Age**		
**4. Monthly household income (RMB)**			Below 19	105	9.8
Below 3,000	378	35.4	20–29	374	35.1
3,001–5,000	326	30.6	30–39	309	29.0
5,001–7,000	237	22.2	40–49	192	18.0
7,001–9,000	76	7.1	50–59	67	6.3
Above 9,000	37	3.5	Above 60	19	1.8
Missing	13	1.2	Missing	1	0.1

## Results

We used AMOS 24.0 to analyze the data as AMOS is the most commonly used software for estimating covariance-based structural models. We have a large enough sample and the variables are normally distributed, so AMOS is suitable for our data. A two-stage procedure was applied to analyze the data. In the first stage, the measurement model was assessed in terms of its reliability and validity. In the second stage, the structural model was examined. The significance of the model estimates was based on a bootstrapping procedure with 5,000 samples.

### Reliability and Validity Test

The confirmatory factor analysis (CFA) was used to measure the reliability and validity of the data. The initial CFA results show that the CFA fit indices represented an appropriate model fit (*x*^2^ = 928.575, df = 224, *x*^2^/df = 4.145, CFI = 0.922, AGFI = 0.903, IFI = 0.923, GFI = 0.928, RMSEA = 0.054). Further, we measured the convergent and discriminant validity to ensure data validity. The value of Cronbach’s α ranges from 0.704 to 0.831 which is well above the acceptable limit of 0.7, suggesting good internal reliability among the items of each construct. Factor loading and average variance extracted (AVE) were used to test the convergent validity. After deleting “I plan to buy organic food in the next two weeks” and “there is too much organic food information for me to make a reasonable choice” with low factor loading, the factor loading of all items is above 0.5, which meets the criterion. The value of AVE was above or close to 0.5. The composite reliability (CR) values of each construct exceeded the threshold of 0.60 (from 0.682 to 0.831), showing an ideal internal consistency among the construct items. Please refer to Table 2 for the detailed results of reliability and convergent validity.

**TABLE 2:** Results of confirmatory factor analysis (CFA).

Construct	Items	Loading	CR	Cronbach’s α
Egoistic values	Authority: the right to lead or command	0.505***	0.682	0.738
	Social power: control over others, dominance	0.622***		
	Wealth: material possessions, money	0.661***		
	Influential: having an impact on people and events	0.572***		
Altruistic values	Social justice: correcting injustice, care for the weak	0.455***	0.685	0.708
	Helpful: working for the welfare of others	0.634***		
	Equality: equal opportunity for all	0.731***		
	A world at peace: free of war and conflict	0.541***		
Health Concern	I chose food carefully to ensure the good health	0.722***	0.688	0.704
	I didn’t consider myself as health conscious Consumer	0.701***		
	I think often about health related issues	0.523***		
Environmental Concern	The balance of nature is very delicate and can be easily upset	0.792***	0.741	0.741
	Human beings are severely abusing the environment	0.786***		
	Humans must maintain the balance with nature in order to survive	0.565***		
	Human interferences with nature often produce disastrous consequences	0.407***		
Purchase Intention	I am glad to buy organic foods	0.752***	0.794	0.822
	I expect to consume organic foods	0.791***		
	I would buy organic food products	0.710***		
	I plan to consume organic foods	0.534***		
	I intend to purchase organic foods produce within the next 2 weeks	NA		
Perceived uncertainty	1. I’m not sure of my knowledge about organic foods	0.735***	0.799	0.831
	2. I’m not confident of my personal view on organic foods	0.765***		
	3. Organic food labels lead me to be unsure of the best choice for me	0.660***		
	4. There is too much organic product information for me to make the right choice	NA		
	5. I have no confidence in evaluating between organic foods and conventional foods	0.609***		
	6. I’m not confident of those organic foods in the current market	0.546***		

***p < 0.001.

Finally, the discriminant validity was evaluated. The square root of the AVE of each construct was higher than its correlation value. In addition, Brown (2006) suggested that to ensure the validity of discrimination, the correlation value between all constructs should be less than 0.8. Please refer to Table 3 for the detailed results of discriminant validity.

**TABLE 3:** Correlations of variables.

	EV	AV	HC	EC	PI	PU
**EV**	***0.593***					
**AV**	0.362***	***0.599***				
**HC**	0.192***	0.380***	***0.655***			
**EC**	0.651***	0.288***	0.243***	***0.658***		
**PI**	0.396***	0.501***	0.465***	0.378***	***0.704***	
**PU**	0.337***	–0.087*	0.070	0.397***	–0.091*	***0.668***

*EV, egoistic value; AV, altruistic value; HC, health concern; EC, environmental concern; PI, purchase intention; PU, perceived uncertainty.*

*The square roots of AVE for discriminant validity are italicized along the diagonal.*

*p < 0.05; ***p < 0.001 (two-tailed).

### Hypotheses Testing

#### Structural Equation Model

Table 4 shows the results based on Structural equation model (SEM). The value of β represents the association between the independent variable and dependent variable. It can be seen that the altruistic value significantly influences the EC (β = 0.401, *t* = 7.823, *p* < 0.001) and organic purchase intention (β = 0.277, *t* = 5.782, *p* < 0.001). Therefore, the hypotheses H1b and H2b were supported. The egoistic value significantly influences the HC (β = 0.198, *t* = 4.654, *p* < 0.001) and purchase intention (β = 0.145, *t* = 3.446, *p* < 0.01). Therefore, the hypotheses H1a and H2a were supported. Finally, HC has a significant influence on the purchase intention (β = 0.283, *t* = 7.559, *p* < 0.001) and EC has a significant influence on the purchase intention (β = 0.163, *t* = 4.170, *p* < 0.001), which supported the Hypotheses H3a and H3b.

**TABLE 4:** Path analysis of structural equation model (SEM).

Path	β	t-Statistics	Sig.	Hypothesis
Egoistic value→Health concern	0.198	4.654	***	H1a supported
Altruistic value→Environmental concern	0.401	7.823	***	H1b supported
Health concern→Purchase intention	0.283	7.559	***	H3a supported
Environmental concern→Purchase intention	0.163	4.170	***	H3b supported
Egoistic value→Purchase intention	0.145	3.446	***	H2a supported
Altruistic value→Purchase intention	0.277	5.782	***	H2b supported

***p < 0.001 (two-tailed).

#### Test of Mediation Effect

The PROCESS program for SPSS (model 4) is used to test the role of HC and EC as mediators between the egoistic value, altruistic value, and purchase intention, respectively (Preacher and Hayes, 2008). Bootstrapping was performed at a 95% CI with 5,000 bootstrap samples to investigate the indirect effects of the independent variable through a mediator. The CI of the lower and upper bounds was calculated to test whether the indirect effects were significant. As seen in Table 5, in both the cases, HC and EC show a partial mediation effect. HC partially mediates the relationship between the egoistic value and purchase intention (an indirect effect = 0.044), and EC partially mediates the relationship between the altruistic value and purchase intention (an indirect effect = 0.077) of organic foods. Thus, both H4a and H4b are supported.

**TABLE 5:** The results of the mediating effect analysis.

	Direct effect				Indirect effect		
Path	Effect	LLCI	ULCI	*P* value	Effect	LLCI	ULCI
EV→HC→PI	0.278	0.220	0.337	0.000	0.044	0.021	0.071
AV→EC→PI	0.334	0.280	0.398	0.000	0.077	0.053	0.104

*EV, egoistic value; AV, altruistic value; HC, health concern; EC, environmental concern; PI, purchase intention.*

#### Test of the Moderation Effect

**FIGURE 2:**
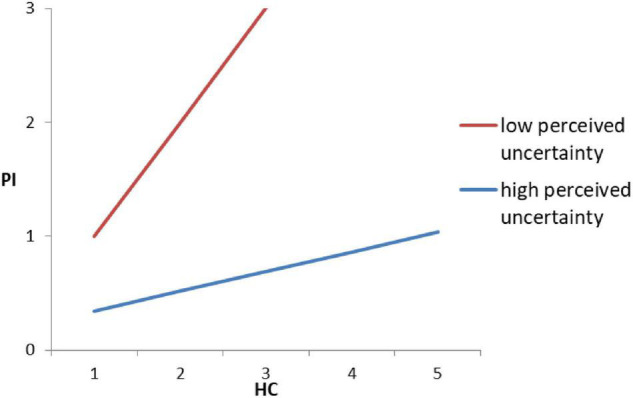
Two-way interaction of perceived uncertainty and health concern (HC).

We used hierarchical multiple regression to test the moderating role of perceived uncertainty. When using HC as an independent variable, the interaction of HC and uncertainty is significantly negative (*B* = −0.102, *p* < 0.001), implying that perceived uncertainty plays a negative moderating role in the relationship between HC and organic purchase intention, which supports H5a. The finding indicates that the consumer perceived uncertainty will weaken a positive relationship between HC motivation and organic purchase intention. That is, the stronger the perceived uncertainty of consumers for organic food, the weaker the promotion effect of HC motivation on the organic food purchase intention. The two-way interaction effects are shown in Figure 2.

**FIGURE 3:**
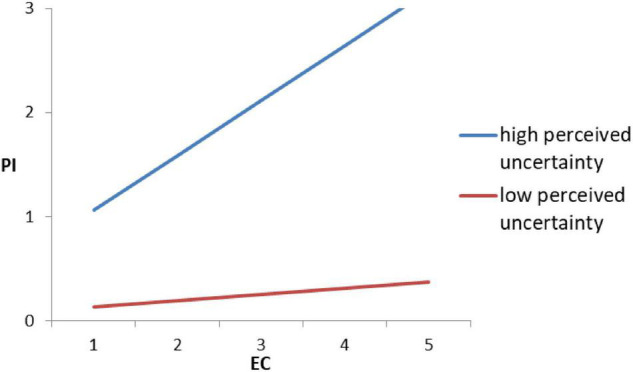
Two-way interaction of perceived uncertainty and environmental concern (EC).

When using EC as an independent variable, the interaction of EC and uncertainty is significantly positive (*B* = 0.134, *p* < 0.001), implying that perceived uncertainty plays a positive moderating role in the relationship between EC and organic purchase intention, which is contrary to the hypothesis H5b. That is, perceived uncertainty will strengthen the influence of EC motivation on the purchase intention of organic foods. The stronger the perceived uncertainty of consumers for organic food, the stronger the promotion effect of EC motivation on the purchase intention of organic foods. The two-way interaction effects are shown in Figure 3.

## Discussion

### Theoretical Implications

This study aims to investigate and understand the significance of altruistic and egoistic values in determining consumers’ willingness to buy organic foods in the context of China, which is the largest developing nation in the world. The findings report that both altruistic and egoistic values play a significant role in determining the motives and intention toward purchasing organic foods, which verifies the significance of stable value orientations in explaining people’s consumption intention of organic foods (Chen, 2020). Specifically, the altruistic value is found to influence the consumer’s EC, and the egoistic value is found to influence the consumer’s HC. The finding is consistent with Yadav (2016) as they report that both EC and egoistic HC significantly influence the attitude and intention toward purchasing organic foods among Indian youth. In addition, it is found that, compared to EC, the HC has a stronger impact on the consumer’s intention of buying organic foods. The findings support the study of Magnusson et al. (2003) and Yadav (2016) that when consumers decide to buy organic foods, the egoistic motive is more influential compared to an altruistic motive.

The results of this empirical study in the context of China confirm the previous findings (e.g., Yiridoe et al., 2005; Vermeir and Verbeke, 2006; Schleenbecker and Hamm, 2013; Hemmerling et al., 2015; Teng and Lu, 2016; Ahangarkolaee and Gorton, 2020; Nafees et al., 2022) that the consumer’s health consciousness, food safety concerns, and ecological motives, which are the subdimensions of organic consumption motives and are all positively related to involvement with organic foods. Specifically, a consumer with HC is more likely to take health-related behavior and consume organic foods, which are related to their internal needs for health and safety (Hill and Lynchehaun, 2002; Bezençon and Blili, 2010; Michaelidou and Hassan, 2010).

Furthermore, those who are conscious of environmental protection and animal welfare are more involved in organic food consumption, which is consistent with some previous results (e.g., Chen, 2007; Hjelmar, 2011; Teng and Lu, 2016) but is also inconsistent with other findings (e.g., Zakowska-Biemans, 2011; Zagata, 2012). Zakowska-Biemans (2011) also mention that although people widely recognize the importance of environmental protection and animal welfare, it is not relevant to the organic purchase decision. Nevertheless, researchers claim that the consumers with ecological motives are more involved in an ethical product decision (Bezençon and Blili, 2010), which further has a positive impact on consumers’ intention of purchasing organic foods. In this research, the finding of a positive effect of ecological motives reconfirms the results of Chen (2007), Teng and Lu (2016), and Zhu (2018), indicating that consumers in China pay attention to environmental protection in their daily food intake, so they are more likely to consume organic foods related to environmental values.

In addition to the effects of consumption motives on consumers’ willingness to buy organic foods, this study also confirms the mediating effect of consumption motives on the relationship between altruistic values, egoistic values, and organic purchase intention. The results show that consumption value and consumption motivation are two different concepts. Mixing consumption motivation with values (such as Yadav, 2016) prevented us from understanding the complete mechanism of value-motivation-behavior. In fact, our research has proven that the values rooted in an individual’s heart affect the purchase behavior of organic foods through driving consumption motivation. Whether in the relationship between egoistic values or altruistic values and organic food purchase intention, the mediating effect of EC is stronger than that of HC. In the past, many studies have concluded that the effect of HC motivation is greater than that of EC motivation (Magnusson et al., 2003). The possible reason for this result is that with a continuous deterioration of the environment, people increasingly feel that their survival and development are under a direct threat. Therefore, the awareness and willingness of environmental protection of Chinese consumers are gradually increasing, which makes the EC motivation play an increasingly important role.

This study also found that perceived uncertainty significantly weakens the positive impact of HC on the purchase intention of organic foods. Such a result shows that even though buying organic food is normally considered better for health than conventional foods, feeling a strong uncertainty would hinder the influence of HC on the purchase decision of organic foods. This is because the perceived risk caused by incomplete information and uncertainty may have a negative effect on consumer purchase intention (Hassan et al., 2013). When consumers feel uncertain about the consequences of eating organic foods, they are unlikely to make a decision to buy organic foods because they do not possess the relevant knowledge and information to accurately predict the outcome of organic food consumption (Thøgersen, 2009; Chen and Huang, 2013).

Surprisingly, this study found that perceived uncertainty significantly weakens the influence of EC on the intention of organic purchasing, which shows that a strong sense of uncertainty would promote the influence of EC on the purchase decision of organic foods. The higher the perceived uncertainty, the stronger the impact of EC on the intention of purchasing organic foods. In other words, the lower the perceived uncertainty, the weaker the influence of EC on the intention of purchasing organic food. In terms of the main effect, the motivation to care about environmental protection urges consumers to think about the benefits of organic foods to the environment. When they confirm that organic food planting is beneficial to environmental protection (such as less use of pesticides and fertilizers), they are more willing to buy organic foods. However, the lower the feelings of uncertainty about organic foods, that is, the higher the feelings of certainty, the weaker their willingness to consume organic foods. This may be that the more consumers think that they have related knowledge about organic foods (note that this knowledge may include not only the health benefits of organic foods, but also the negative effects of organic foods on the environment), the more they doubt the benefits of organic food planting on environmental protection, so it weakens the influence of environmental motivation on the willingness to purchase organic foods. Chinese consumers’ cognition of organic foods mainly focuses on health and taste because in most Chinese people’s understanding, organic cultivation is actually the traditional agricultural cultivation method (such as using human and animal excrement as a fertilizer), and the traditional planting method also has a certain negative impact on the environment. In fact, whether organic agriculture is good or bad for the ecological environment is still a controversial topic in the scientific community. Therefore, when consumers’ perception of organic foods is uncertain, they usually follow the mainstream cognition of society, that is, organic agriculture does not use chemically synthesized pesticides and fertilizers, which are conducive to an ecological balance. However, when consumers learn more about the process of organic food cultivation, they may find the disadvantages of organic agriculture, such as the need for more arable land due to low yield, thus reducing the forest area.^3^

### Practical Implications

The findings of this study are conducive to guiding the development of the organic food industry. Some management implications and suggestions from the results of this research are as follows.

According to this study, two kinds of consumption motives have positive influences on consumers’ willingness to buy organic foods. Moreover, the two values have positive influences on the related consumption motives, and then have a positive impact on the intention of organic food consumption. The empirical results show that marketers in the organic food section should understand the needs, values, and benefits of organic foods perceived by consumers with each consumption motive in terms of health and environmental aspects with the aim to make effective marketing communication strategies. Specifically, marketers should use the most authoritative scientific evidence to emphasize the health and safety of organic foods. If consumers feel more that organic foods can provide health and safety benefits to meet their health needs, they will become more willing to consume organic foods. Similarly, marketers should let consumers with an environmental motivation understand that the production and packaging of organic foods are carried out in a way that protects the environment (Lockie et al., 2002). In this way, consumers can be confident that organic food production strictly abides by environmental principles, which will strengthen their organic purchasing behavior. Marketing personnel should aim to improve consumers’ awareness and knowledge of organic products to promote consumption (Marreiros et al., 2021).

Besides, the results show that perceived uncertainty has a negative moderating effect on the relationship between the consumer’s health motives and the purchase intention of organic products. As the perceived uncertainty comes from consumers’ incomplete information and risk perception in the process of transaction, marketers should convince consumers by providing reliable information, so as to enhance consumers’ confidence in the health benefits of organic foods. The literature shows that to improve the ability of consumers to verify the attributes and values of organic foods publicized by organic food suppliers, it is necessary to establish public trust in the organic food certification system and organic food traceability system (Janssen and Hamm, 2011). Although the food traceability system is widely used in the organic food industry to reduce the information asymmetry between consumers and suppliers (Chen and Huang, 2013), the traditional food traceability system is difficult to ensure its objectivity and fairness, so the latest blockchain technology should be introduced. Blockchain food traceability system has the characteristics of full transparency, traceability tracking, and tamper proof, which can ensure the authenticity of the information. As China’s organic market is still in the development stage, it is very important to establish a reliable organic food system and provide reliable and comprehensive information for improving consumers’ confidence in organic foods.

Besides, the results indicate that perceived uncertainty has a positive moderating effect on the relationship between consumer environmental motives and the intention of organic purchase. This means that a strong sense of uncertainty toward organic foods positively moderates the influence of EC on consumers’ willingness to buy organic foods. Based on the aforementioned discussion, we believe that the agricultural sector needs to improve the environmental attributes of organic agriculture. In the process of developing from traditional organic agriculture to industrialized organic agriculture, practitioners should not only use scientific and technological means to ensure and strengthen the health and environmentally friendly attributes of organic foods but also reduce and eliminate the adverse factors on the environment in the process of organic agriculture planting, so that organic agriculture can truly evolve into green organic agriculture.

### Limitations

This study inevitably has some limitations. First, this study does not consider other values such as biosphere value and Confucian value, which were found to influence pro-environmental behavior (Shin et al., 2017; Zhang et al., 2020). Second, this study did not conduct random sampling during the survey, which makes the representativeness of the sample insufficient. In the future, if we want to reveal Chinese consumers’ willingness to buy organic foods, it is necessary to carry out a large-scale random sampling survey. Finally, the self-report method used in this survey has its inherent defects. In the future, laboratory research methods can be considered to reveal and verify the impact mechanism of organic food consumption.

## Data Availability Statement

The raw data supporting the conclusions of this article will be made available by the authors, without undue reservation.

## Ethics Statement

Ethical review and approval was not required for the study on human participants in accordance with the local legislation and institutional requirements. Written informed consent for participation was not required for this study in accordance with the national legislation and the institutional requirements.

## Author Contributions

SW: conceptualization, data curation, project administration, and supervision. FL: formal analysis and methodology. SS: investigation. SS and FL: Writing—original draft. SS and RW: writing—review and editing. All authors contributed to the article and approved the submitted version.

## Conflict of Interest

The authors declare that the research was conducted in the absence of any commercial or financial relationships that could be construed as a potential conflict of interest.

## Publisher’s Note

All claims expressed in this article are solely those of the authors and do not necessarily represent those of their affiliated organizations, or those of the publisher, the editors and the reviewers. Any product that may be evaluated in this article, or claim that may be made by its manufacturer, is not guaranteed or endorsed by the publisher.

## Footnotes

1
https://www.360kuai.com/pc/92f35c2eb952fd0b4?cota=4&kuai_so=1&tj_url=so_rec&sign=360_57c3bbd1&refer_scene=so_1

2
http://www.xinhuanet.com/fortune/2018-08/21/c_1123299476.htm

3 Impact of organic agriculture on climate change. http://news.sciencenet.cn/sbhtmlnews/2019/1/342478.shtm?id=342478
